# Notch Inhibitors and BH3 Mimetics in T-Cell Acute Lymphoblastic Leukemia

**DOI:** 10.3390/ijms252312839

**Published:** 2024-11-29

**Authors:** Ilaria Sergio, Claudia Varricchio, Federica Squillante, Noemi Martina Cantale Aeo, Antonio Francesco Campese, Maria Pia Felli

**Affiliations:** 1 Department of Experimental Medicine, Sapienza University of Rome, 00161 Rome, Italy; ilaria.sergio@uniroma1.it (I.S.); federica.squillante@uniroma1.it (F.S.); 2 Department of Molecular Medicine, Sapienza University of Rome, 00161 Rome, Italy; claudia.varricchio@uniroma1.it (C.V.); noemimartina.cantaleaeo@uniroma1.it (N.M.C.A.); antonello.campese@uniroma1.it (A.F.C.)

**Keywords:** Notch, Bcl-2 family proteins, T-cell leukemia, BH3 mimetics, Notch inhibitors

## Abstract

T-cell acute lymphoblastic leukemia (T-ALL) is an aggressive hematological malignancy with poor response to conventional therapy, derived from hematopoietic progenitors committed to T-cell lineage. Relapsed/Refractory patients account for nearly 20% of childhood and 45% of adult cases. Aberrant Notch signaling plays a critical role in T-ALL pathogenesis and therapy resistance. Notch inhibition is a promising therapeutic target for personalized medicine, and a variety of strategies to prevent Notch activation, including *γ*-secretase (GS) inhibitors (GSIs) and antibodies neutralizing Notch receptors or ligands, have been developed. Disruption of apoptosis is pivotal in cancer development and progression. Different reports evidenced the interplay between Notch and the anti-apoptotic Bcl-2 family proteins in T-ALL. Although based on early research data, this review discusses recent advances in directly targeting Notch receptors and the use of validated BH3 mimetics for the treatment of T-ALL and their combined action in light of current evidence of their use.

## 1. Introduction

The most common subtype of leukemia is acute lymphoblastic leukemia (ALL), which accounts for 80% of all cases. B-ALL or T-ALL are severe malignant hematopoietic disorders originating from the uncontrollable clonal expansion of B or T precursor cells, respectively. T-ALL is less frequent but more aggressive than B-ALL [1,2] and originates from the thymus [3]. There is an unmet need to improve therapies for T-ALL, given the high rate of relapse and poor outcomes, particularly in adults [1].

Accumulating genetic aberrations during T-cell maturation alters the control of cell growth, proliferation, survival, and differentiation. Notch1 mutations are present in 50–70% of T-ALL cases [4,5] and the vast majority have increased expression and function of Notch3, but rare mutations have been described [6]. Notch3 activating mutations without Notch1 activation have been detected in a T-ALL patient cohort [6], suggesting the pathogenic role of either Notch1 or Notch3 activation in this disease. Considering that deregulated Notch signaling has been reported to contribute to chemotherapy resistance [7], targeting Notch signaling presents challenges for therapeutic strategies.

**Figure 1:**
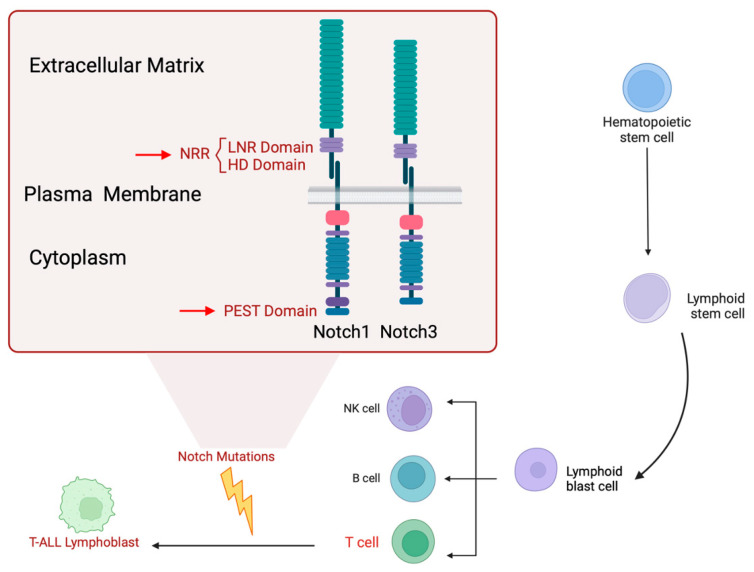
Notch receptors domains and key mutation sites. In T-ALL lymphoblast activating mutations of Notch1 and Notch3 receptors are frequently detected. Notch1 extracellular N-terminal domain N-ECD contains 36 while Notch3 34 homologous (EGF)-like repeats, respectively (green). The NRR is composed of three LNR (lilac) and the juxta membrane HD domains that hold the Notch heterodimer and keep the receptor in a resting place. RAM is a RBPJ-associated molecule domain (pink). NLS, nuclear localization signal (pale violet); ANK, ankyrin repeats (blue); TAD, transactivation domain (intense violet) lacking in Notch3 receptor; and PEST. Mutations are frequently detected in NRR (arrow), while in 12.5% when localized in PEST domain (arrow), a peptide sequence rich in proline, glutamate, serine and threonine, that contains degradation signals and is critical for NI-CD stability. HD and PEST mutations in 17.7% of T-ALL cases [4,29]. Abbreviations: EGF, Epidermal growth factor; NRR, Negative Regulatory Region; LNR, cysteine-rich Lin12-Notch repeats; HD, Heterodimerization Domain; PEST, Proline/glutamic acid/serine/threonine-rich motifs domain; NI-CD, Notch intracellular domain. Created in Biorender.com.

Different genetic mechanisms target Notch genes, ranging from missense mutations to in-frame deletions or insertions in different functional domains of the receptor (Figure 1). Notch1 mutations are essentially located in the heterodimerization domain (HD) (40–50% of T-ALL samples) [4], or in the LIN12-Notch repeats (LNR) domain in the negative regulatory region (NRR) in the extracellular region of the receptor [8]. Indeed, the heterodimerization (HD) domain mutations in exons 26 and 27, which expose the S2 site to cleavage in a ligand-independent manner by ADAM-type metalloproteases, impair Notch1 heterodimer stability (class I HD mutants). Alternatively, in class II HD mutants, short insertions (14 bp) displace and expose the ADAM site-2, allowing for constitutive ligand-independent Notch1 activation [9]. Insertions to expand the juxta membrane region (JME mutants) have been also described [10]. Mutations clustering at the C-terminal aminoacidic sequence of Notch1 leads to premature stop codon generation and results in PEST degron motif deletion (exon 34), thus impairing Notch1 intracellular domain (ICD) degradation [7]. Additionally, PEST mutations can compromise the recognition by FBXW7, an E3 ubiquitin ligase that targets Notch for proteasomal degradation and increases Notch stability. This stability can also be increased by FBXW7 gene mutations, detected in 20% of T-ALL cases, and associated with resistance to γ-secretase (GS) inhibitors (GSIs) [10,11]. Additionally, high expression of USP7, which deubiquitinates and stabilizes Notch1, is found in T-ALL [12]. Activating mutations of Notch1 in the NRR and PEST domains leads to constitutive activation of Notch1 signaling and triggers the onset of T-ALL [13]. Among the mutations described for Notch1, only those driving to constitutive activation promote a strong Notch1 signaling that induces T-ALL in murine models [14]. Despite Chronic Lymphocytic Leukemia (CLL) patients, non-coding mutations in the Notch1 3’UTR have never been reported [15]. However, Notch3, another member of the Notch receptor family, has shown a well-established T-cell oncogenic capacity in a mouse model [16,17,18], and its mutation has been found in infant T-ALL [19]. Interestingly, activating mutations in NRR and PEST domains of Notch3 have been described in several tumor cell lines, including an NRR mutation in a human TALL1 cell line that likely makes the S2 site more accessible to ADAM metalloproteases [6]. These authors also described, for the first time, the presence of Notch3 activating mutations in two out of forty T-ALL primary samples and in twelve out of twenty-four patient-derived orthotopic T-ALL xenograft models, two of which showed activation only of Notch3. Therefore, either Notch1 or Notch3 activation can support the pathogenesis of T-ALL. In this context, several direct Notch target genes, such as MYC, HES1, and IL7R, have been shown to induce and maintain T-ALL [20].

Notch activation is an early hallmark in T-cell leukemogenesis and a key regulator of the leukemia-initiating cells (LICs) in “Notch-addicted” T-ALL [8,21].

The thymus is the perfect location for the interplay of multiple molecular and cellular mechanisms that drive normal T-cell growth and differentiation [22], and, as mentioned above, is crucial for T-ALL development. The B-cell lymphoma (Bcl-2) gene is highly expressed in CD34 positive (CD34^+^) T-cell progenitors and gradually decreased during differentiation with the lowest value, also for the protein, in CD4^+^CD8^+^ Double Positive (DP) T-cells [23], which are included, according to the immunologic classification of T-ALL, in the third subgroup, which contains cortical T-cells. Bcl-2 family members are downstream targets of the IL7R/Jak/STAT pathway that are aberrantly activated in T-ALL [24]. Interestingly, Bcl-2L1 (Bcl-xL) expression showed an inverse trend, which has been confirmed also at the protein level, being higher in DP T-cells. Notably, the high expression of antiapoptotic Bcl-2 is a hallmark of immature subtypes of T-ALL, which reflects the spatiotemporal regulation of Bcl-2 during normal T-cell development [25]. Moreover, a similar level of the antiapoptotic Bcl-2 is also detected in the HOXA-positive T-ALL subgroup, whereas mature T-ALL is often characterized by TAL1 or Lmo2 oncogene activation, displaying low levels of Bcl-2 [23]. Besides the role of Bcl-2 as a Notch1 target gene in solid tumors [7] and in B-CLL [26], Notch3 can trigger the transcription of a Bcl-2 family member, Bcl-2A1, in a Notch-dependent T-ALL model [27]. Moreover, in a zebrafish model of human Notch1-induced T-ALL, stable overexpression of Bcl-2 dramatically facilitates Notch1-mediated leukemia onset, indicating the synergy between the Notch pathway and the Bcl-2-mediated anti-apoptotic pathway [28]. This model could reveal mutant genes that could either promote or delay the onset of leukemia and that could serve as candidate targets for new therapies.

Given the crucial role played by Notch signaling and Bcl-2 expression in T-ALL development and progression, as well as the pressing need for novel treatments in relapsed/refractory (R/R) cases, we will explore, in the present review, the role of single treatments in the context of T-ALL and the current knowledge about their combined effect, giving some insights into how these inhibitors could help to improve responses to conventional therapy.

## 2. Overview of Notch Signaling

**Figure 2:**
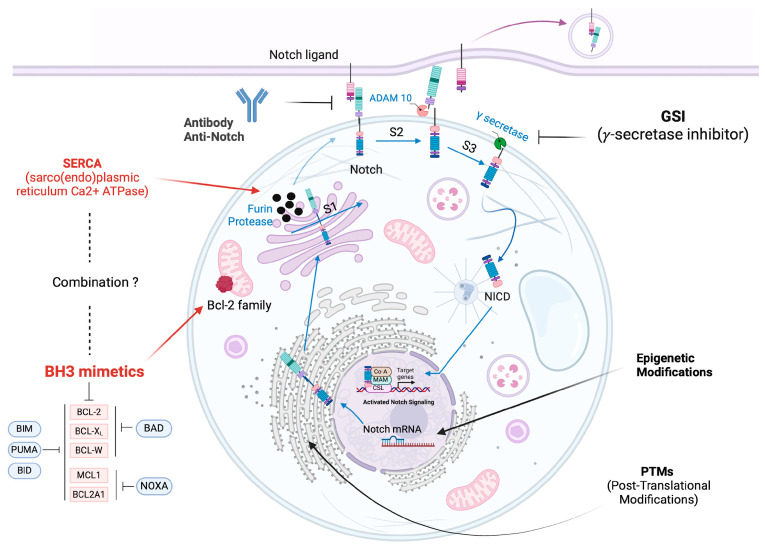
Overview of the Notch signaling pathway with Notch and Bcl-2 therapeutic targets in T-ALL. The Figure depicts the major sites of Notch receptor trafficking as therapeutic sites to contrast Notch signaling by affecting generation and exposition of a furin-cleaved heterodimer of Notch receptor on the cell-surface. Upon ligand binding, the HD domain of the extracellular Notch is exposed to ADAM metalloproteases cleavage. A sequential cleavage by the *γ*-secretase complex at the transmembrane region releases the Notch intracellular domain (NICD). It translocates to the nucleus, binds the transcription factor CSL, recruits p300, Co-A, and Maml, and activates target genes. To shut down the signaling, FBXW7-dependent ubiquitination and proteasomal degradation of the receptor will occur. Notch targeting can be performed at different levels: (1) by inhibiting receptor proteolytic cleavage by *γ*-secretase inhibitors; (2) by blocking ligand-receptor interaction with monoclonal antibodies; (3) by interfering with Notch receptor maturation and trafficking with SERCA inhibitors; (4) by targeting post-translational modification (PTM) to affect receptor functionality; (5) by epigenetic mechanisms to contrast Notch expression. Interactions between initiator BH3 domains (BAD and NOXA) and guardians (Bcl-2, Bcl-xL, Bcl-W, Mcl1, Bcl2A1) and sensitizer BH3-only proteins (BIM, PUMA, BID). The overexpression of guardian proteins enables cancer cells to evade apoptosis. This led to the development of BH3 mimetics that displace guardian-bound executioners (BAX, BAK) or initiators and promote apoptosis [25]. GSI treatment in combination with BH3 mimetics has been already described in the text. A hypothetical new combined strategy may imply SERCA inhibitors as a double hit to tackle T-ALL. Created in Biorender.com.

Multiple signaling pathways can lead to the activation of Notch, whose signaling can be further modulated by numerous intra- and extracellular proteins. Additionally, the processing of the receptor itself is interconnected with its signaling pathway. For this reason, its expression levels influence the effects of the signaling [30]. The Notch gene family is evolutionarily conserved across species, both in terms of receptors and ligands. In vertebrates, there are four Notch genes that encode four single-pass transmembrane receptor proteins (Notch1–4) and two classes of ligands (Delta-like and Jagged) [31]. The receptors are synthesized as single precursor polypeptides that undergo post-translational modifications (Figure 2). Initially, in the Golgi apparatus, the receptor is cleaved at the S1 site by the convertase Furin protease, generating a heterodimeric conformation (Figure 2). Subsequently, interaction with the ligand (DSL) expressed on the surface of adjacent cells triggers two proteolytic cleavages of Notch. The first is mediated by a metalloprotease (ADAM10 or ADAM-17) at the S2 site [32], which exposes the extracellular domain for endocytosis by the adjacent cell. The active intracellular domain is then released into the cytoplasm following a second proteolytic cleavage at the S3 site by γ-secretase [33]. The two proteolytic events release the active ICD of the receptor, which enters the nucleus, associates with the DNA-binding factor RBPJK, and recruits co-activators from the Mastermind-like (MAML) family to activate the expression of target genes in a canonical or non-canonical manner [34].

The canonical Notch signaling activates different pathways, including NF-kB, that play a key role in essential biological processes such as cell proliferation, maturation, and differentiation [18,35]. This signaling drives multiple cell lineage decisions, including T cell development within the thymus [36]. As mentioned above, Notch alterations are a crucial event in T-ALL pathogenesis. Although intensive chemotherapy improved overall survival outcomes, R/R cases remain particularly challenging to treat. Approximately 20% of pediatric and 40% of adult patients experience a recurrence of the disease [37]. Additionally, since Notch signaling has been implicated in chemotherapy resistance, there is the unmet need to combat the progression of T-ALL.

## 3. Notch Inhibitors

In this review, we aim to illustrate the various strategies under development to directly or indirectly inhibit Notch receptors: *γ*-secretase inhibitors (GSI); monoclonal antibodies (mAbs); and sarco(endo)plasmic reticulum Ca^2+^ ATPase (SERCA) inhibitors.

### 3.1. Gamma Secretase Inhibitors (GSIs)

As previously mentioned, *γ*-secretase is an intramembrane protease crucial for the third cleavage of Notch receptors and the release of the active Notch Intracellular Domain (NICD) (Figure 2). It is composed of two stable subunits, nicastrin and presenilin enhancer, and two variable subunits, PSEN1/2 and APH1A/B. The different combinations of these subunits result in the formation of four distinct sub-complexes. Small molecules known as *γ*-secretase inhibitors (GSIs) primarily target the PSEN1/2 subunits located within the catalytic core [38]. The key function of these inhibitors is to modulate Notch signaling through their pan-Notch inhibitory activity, preventing its nuclear translocation and subsequent activation of target genes. GSIs are used as anticancer agents, particularly in contexts where Notch plays an oncogenic role, such as in T-ALL, both in vitro [4] and in vivo [39,40].

Several studies have suggested that GSI treatment in T-ALL cell lines with activating Notch1 mutations reduces the levels of the active protein and downregulates Notch target genes, leading to G1 cell cycle arrest and varying degrees of apoptosis [41]. However, some cell lines have exhibited resistance to GSI due to mutations in PTEN and FBXW7, both of which sustain leukemic cell proliferation despite Notch inhibition [7]. According to preclinical studies, GSI could be a promising treatment, but one of the challenges associated with the drug is its lack of specificity for an individual Notch receptor.

Additionally, demonstrating its role in the gut, immune system, and skin homeostasis, severe adverse events such as gastrointestinal toxicity, skin disorders, and diarrhea are observed in patients receiving chronic oral GSI treatment [42] due to the simultaneous inhibition of both Notch1 and Notch2.

Although GSI is not widely used in clinical practice, new GSI compounds that specifically modulate this enzymatic activity have been introduced in clinical trials and preclinical studies. Targeted pathways and their side-effects are delineated in Table 1.

GSI monotherapy may upregulate alternative pathways and compensatory mechanisms such as kinase or growth factor pathways, allowing cells to continue proliferating or evade apoptosis and thus developing resistance (or insensitivity) to *γ*-secretase inhibition. Indeed, in GSI-resistant T-cells, there is a reduced expression of PTEN due to the upregulation of the PI3K/AKT pathway compared to GSI-sensitive NOTCH1-dependent T-ALL cells [49]. The rationale for combining GSI with other drugs may stem from expression-based virtual screening to identify highly active drugs, such as withaferin A, which has the strongest cytotoxic and GSI-synergistic antileukemic effects in vitro and in vivo, or the potential of proteomics to dissect alterations in cellular signaling and identify druggable pathways [50]. Moreover, based on GSI’s limited antitumor effects, due to transient growth arrest rather than cell death, an alternative option could include the use of BH3 mimetics, which is known to promote apoptosis, to enhance the overall efficacy of T-ALL treatment.

### 3.2. Antibodies to Contrast Notch Signaling

Notch inhibitory antibodies have been developed as an alternative approach to overcome the modest efficacy, questionable selectivity, and toxicity associated with pan-Notch inhibition by GSIs (Figure 2). Two types of Notch-targeting antibodies have been developed and are distinguished by their mechanisms of action: antibodies that counteract the conformational change after binding to the ligand and antibodies that directly block the ligand binding.

The first type comprises Notch inhibitory mAbs that block protein-cutting and signaling. As we know, Notch binding to its ligand results in a conformational change in the receptor’s negative regulatory region (NRR), i.e., Notch’s activator ‘button’. This change allows exposure of the S2 juxta membrane site and recognition/cutting by the protease. The antibodies developed in this way have an inhibitory mechanism based on stabilizing the quiescence of the NRR, i.e., the conformation in which the S2 site is buried, and subsequent blocking of proteolytic cutting by ADAM-10 [51]. In T-ALL cell lines, anti-NRR antibodies can inhibit Notch1 with class I mutations, leading to a significant reduction in cell growth [52]. However, Notch1 receptors with different NRR mutations appear to be completely resistant to this approach. This mechanism may be favored for targeted therapy of common type NRR mutations [51]. The most tolerable antibody is the anti-NOTCH1 mAb OMP-52M51 (Brontictuzumab), which has been evaluated in clinical studies for refractory tumors and R/R lymphoid malignancies (NCT01778439) [53], including T-ALL (NCT01703572) [8]. This mAb also targets Notch1 receptors with activating mutations in the HD/PEST and HD/TAD domains, blocking signaling in response to Jagged1/2 or DLL4 ligands. The effects of anti-NOTCH1 OMP-52M51 have been evaluated in T-ALL cell lines and primary samples [13] and are enhanced when combined with dexamethasone, the main drug of the combined therapy plan used in T-ALL treatment. Besides its effect in prolonging survival, acquired resistance to OMP-52M51 has been described [8,13].

The second type of mAb directly prevents ligand binding. For example, the antibodies Demcizumab (NCT01189968) and Enoticumab (NCT00871559) bind to the DLL4 ligand, thereby blocking the Notch signaling pathway [54]. The Dll4 ligand has been identified as a promising target in tumor angiogenesis in preclinical studies and has been investigated in phase I studies for advanced solid tumors, showing partial responses and disease stabilization [55].

### 3.3. SERCA Inhibitors an Emerging Therapeutic Strategy

As we know, Notch receptor maturation begins in the Endoplasmic Reticulum (ER) through a cleavage at the S1 site by furin-like convertase (Figure 2). This process leads to the formation of a heterodimer, whose structural integrity requires the accumulation of intracellular Calcium (Ca^2+^), mediated by the SERCA pump (sarco(endo)plasmic reticulum Ca^2+^ ATPase). The genes ATP2A1, ATP2A2, and ATP2A3 encode for SERCA1, 2, and 3, respectively [56], proteins that belong to the superfamily of active transporters known as ATPases, located in the ER. More than 70 SERCA isoforms are known, resulting from alternative splicing [57], sharing up to 85% sequence homology but differing in tissue distribution and Ca^2+^ binding affinity in both normal and tumor tissues [58]. This explains their non-redundant role in physiological processes and human diseases [59].

Thus, the role of SERCA pumps is to regulate intracellular Ca^2+^ homeostasis for the trafficking of membrane proteins, including Notch receptors, while a loss of Ca^2+^ leads to impaired maturation [58].

SERCA inhibitors have been identified as potential regulators of mutant NOTCH1 signaling associated with leukemogenesis, functioning by modulating cell cycle progression, and the expansion of T-ALL xenograft models [58]. Binding with Thapsigargin inhibits the enzyme in a Ca^2+^-free state, blocking activation of the catalytic cycle [60]. At this stage, misfolded Notch1 receptors are retained in the ER, leading to a reduction in surface levels and thereby inhibiting signaling and cellular growth in T-ALL [58]. Since SERCA is expressed in all tissues, this lack of specificity poses challenges, which is why different inhibitors have been developed with the goal of reducing off-target toxicity:

Casearin J (CJ): A tricyclic clerodane diterpene (TCD), Casearin J, derived from *Casearia sylvestris*, targets SERCA. CJ reduces Notch1 cell surface expression and prevents the generation of cleaved NICD1 molecules, resulting in decreased signaling and downregulating MYC and HES1 expression in human T-ALL cells. CJ is more active against HD-mutated T-ALL cells but its role on Notch1 WT is not ruled out [61].

Curcumin: Known as diferuloylmethane, curcumin is a natural polyphenol extracted from the rhizome of *Curcuma longa* and has been tested under various health conditions due to its antioxidant, anti-inflammatory, and antitumor properties [62]. Curcumin binds to SERCA, preventing its interaction with ATP, and has been tested in vivo and in clinical studies for various cancers, though without clear evidence of therapeutic benefits [63,64]. In fact, its role as a potential Notch modulator remains far from being fully established.

Cisplatin: This platinum-based compound, widely used in clinical applications, inhibits both the SERCA pump and the Na^+^/K^+^ ATPase pump simultaneously. Its mechanism of action involves a moderate and compensated release of Ca^2+^ from the ER into the cytosol. Despite the potential risk of Ca^2+^-related toxicity, these compounds are used in clinical applications [65].

CAD204520: It binds to SERCA at a binding groove different from that of Thapsigargin. In preclinical studies, it suppresses mutated Notch1 signaling without causing overt cardiac toxicity. In fact, it reduces contractile efficiency by 25%, representing a mechanical cardiac impairment that is tolerated at a therapeutic concentration in an orthotopic T-ALL in vivo model [66].

Thapsigargin: The Notch1 receptor carrying leukemogenic mutations is more sensitive to this SERCA inhibitor compared to the wild-type receptor. This provides a potential antileukemic therapeutic strategy for T-ALL patients with Notch1 mutations, reducing the gastrointestinal side effects typically associated with pan-Notch inhibition [58]. However, SERCA channels are essential for maintaining intracellular calcium homeostasis across all cell types. Therefore, the direct administration of Thapsigargin to animals or humans could lead to cardiac toxicity because of calcium ion shifts in vivo [58]. Consequently, the development of isoform-specific SERCA inhibitors, particularly those active within the T-ALL microenvironment, is needed. Roti et al. proposed that the nonspecific toxicity of Thapsigargin could be circumvented by conjugating it to folates (FA), which would then be released into T-ALL cells by binding to the folate receptor (FR), absent in normal cells [66]. In this context, “cytotoxic” Thapsigargin could be free to induce apoptosis in malignant cells ([66] references therein). Besides severe cardiotoxicity with Thapsigargin, these compounds display clinical applicability, as for Cisplatin, or tolerable side-effects and efficacy as described in preclinical studies for CAD204520.

### 3.4. Targeting Post-Translational Modification (PTMs) of Notch ICD

Notch proteins undergo various post-translational modifications (PTMs) that regulate their stability and cellular localization [13]. In leukemia, multiple PTMs induce conformational changes in Notch, altering its functionality (Figure 2).

Regarding the Notch1 protein, phosphorylation of the PEST domain in the intracellular domain (ICD) recruits the E3 ubiquitin ligase FBXW7, which is involved in NICD turnover. The deletion of the PEST domain leads to increased NICD stability and protein activity in a cell-context-dependent manner [11]. Although 60% of T-ALL patients present activating mutations in Notch1, this is rarer in Notch3, which is overexpressed even without clear abnormalities, as it is recognized as a target of Notch1 [6].

Notch3 protein is also subject to PTMs, such as phosphorylation of Ser/Thr-Pro motifs. This modification enables interaction with the PPIase domain of Pin1, inducing cis-trans or trans-cis conformational changes around the Ser-Pro or Thr-Pro bond. This results in increased expression of N3-ICD, thereby regulating several oncogenic signals that promote T-ALL [13]. For example, one target of Notch involved in this regulation is the transcription factor NF-kB, which is upregulated by Notch3 in T-ALL [67], or Pin1 activity, which promotes the self-ubiquitination and proteasomal degradation of FBXW7, thus decreasing Notch turnover [68].

Finally, in the T-ALL context, Notch3-ICD undergoes deacetylation by HDAC1, favoring tumor development and progression in a Notch3-induced transgenic mouse model of T-ALL [69]. In line with this, treatment with Trichostatin A, an HDAC inhibitor, shifts the balance toward acetylation by p300, inducing proteasomal degradation of Notch and blocking tumor progression [69].

### 3.5. Epigenetic Modifications for Notch Targeted Therapy

Oncoproteins are influenced not only by genetic mutations but also by epigenetic mechanisms such as DNA methylation, histone modifications, and microRNA regulation (Figure 2). Leukemic cells exploit these processes to enhance the expression of genes that promote T-ALL progression or silence tumor-suppressing genes. For this reason, the “epigenetic machinery” has become an increasingly attractive target in cancer therapies. It has been demonstrated that low levels of DNA methylation are associated with high expression of the Notch3 gene in several T-ALL cell lines and primary samples [70]. This occurs through the binding of Boris/CTCFL to the Notch3 gene promoter, which maintains high levels of tri-methylation of histone H3 lysine 4 (H3K4me3) and low levels of DNA methylation [71,72].

It is known that microRNAs contribute to both functional and maturational regulation of various immune cells, particularly T lymphocytes. Numerous miRNAs are up- or downregulated during thymocyte differentiation and in related diseases, including T-ALL [73]. The miR-17-92 cluster was the first to be identified as involved in T-ALL pathology and includes miR-17, miR-18a, miR-19a, miR-20a, miR-19b-1, and miR-92a. Their mechanism of action in leukemia involves inhibiting certain tumor suppressor genes, such as PTEN, p21, and BCL2L11. Indeed, they are overexpressed in T-ALL samples compared to their T-cell counterparts [74].

As short noncoding RNAs, microRNAs are undoubtedly emerging as biomarkers of cancer diagnosis and drug efficacy prediction [75]. Several studies have revealed that miR-150 expression levels vary during lymphocyte development, and in cell lines, miR-150 is upregulated during the immature stages of T cells [73]. Ghisi et al. reported that miR-150 regulates Notch3 expression during intrathymic development [76]. Moreover, microRNAs, such as miR-19, can be involved in a T-ALL-associated gene rearrangement, including Notch1 locus t(9;14)(q34;q11), thus generating a constitutively active form of Notch1 [8]. Nevertheless, miR223 is a target of Notch but contributes greatly to T-ALL development, either suppressing FBXW7 or targeting ARRB1 (Arrestinb1) to impact on Notch degradative pathways [77,78,79]. Recently, it has been shown that various miRNAs, as candidate biomarkers, are Notch3-regulated in T-ALL, including miR-150, miR-139, and miR-9. Indeed, these miRNAs are upregulated in a preclinical model, leading to abnormal T-cell differentiation to support T-ALL progression [80]. Other miRNAs can exhibit a tumor-suppressive capacity in vitro by inhibiting Notch1 and Notch2 expression in a MYC-dependent manner in the context of T-ALL [81]. The data showed a microRNA-mediated regulatory loop between Notch and MYC.

The interplay between miRNAs and Notch may prove relevant in the pathogenesis of T-ALL, and future examinations in large and well characterized cohorts of patients could help to define its clinical implication. Several criticisms need to be overcome, such as synthetic RNA molecules, advanced delivery technologies, side-effects, and efficacy.

Despite the failure of the first-in-human clinical trial, miRNA-targeting therapies are currently under preclinical and clinical development for the treatment of various diseases and cancers [75], and targeting Notch-related miRNAs represents a promising therapeutic option in ALL malignancies.

## 4. BCL-2 Family Proteins

Bcl-2 was initially recognized as an oncogene associated with follicular lymphoma, a type of cancer that originates from B cells [82]. Later, it was demonstrated that Bcl-2 family proteins regulate mitochondrial apoptosis in response to internal cellular signals, such as DNA damage, oxidative stress, and lack of growth factors [83]. This process is essential for tissue homeostasis by removing damaged cells and preventing the development of cancer [84]. The regulation of this process occurs through the interaction of Bcl-2 family proteins with mitochondria, inducing mitochondrial outer membrane permeabilization (MOMP) [85], which represents the primary event of the apoptotic process.

### 4.1. Structure and Function of Bcl-2 Family Proteins

The Bcl-2 family protein includes approximately 20 elements with distinct functions, classified based on their role and structure (extensively reviewed in [86,87]). They are characterized by the presence of one to four Bcl-2 homology domains (BH1-BH4), which allow them to interact with effector proteins and regulate apoptosis (Figure 2). The proteins can be divided into three main classes:

1. Anti-apoptotic proteins: Bcl-2, Bcl-xL, Mcl1, Bcl-W, and A1. These proteins (guardians) block apoptosis by inhibiting pro-apoptotic proteins, preventing mitochondrial membrane permeabilization and the release of apoptotic factors [88].
2. Pro-apoptotic effector proteins: BAX, BAK, and BOK. These proteins (executioners) promote apoptosis by forming pores in the outer mitochondrial membrane, facilitating the release of cytochrome c, which activates caspases, leading to cell death [89].
3. BH3-only proteins: BAD, BIM, PUMA, and NOXA, which act as sensors of cellular stress. These proteins (initiators) inhibit anti-apoptotic proteins and activate effector proteins [90].

There are two main pathways through which apoptosis can be induced [91]:

- Extrinsic pathway: mediated by death receptors located on the plasma membrane (such as Fas and TNF), which directly activate caspases.
- Intrinsic or mitochondrial pathway: intracellular stress or damage signals induce changes in mitochondria, leading to the release of cytochrome c.

The intrinsic pathway involves Bcl-2 family proteins and operates through the mitochondria, but some models hypothesize that the apoptosome also plays a key role [92]. The mitochondrial model of apoptosis provides that pro-apoptotic proteins like BAX and BAK induce MOMP. These proteins are usually inactive due to inhibitory interactions from anti-apoptotic proteins [93]. When pro-apoptotic proteins receive signals from intracellular sensors, they undergo conformational changes and act by forming pores in the mitochondrial membrane, allowing the release of apoptotic factors, such as Smac/DIABLO (Second mitochondria-derived activator of caspase/direct inhibitor of apoptosis-binding protein with low pI), AIF (Apoptosis-Inducing Factor), and cytochrome c. Once in the cytoplasm, cytochrome c interacts with APAF-1 (Apoptotic Protease-Activating Factor-1) and procaspase-9 to form the apoptosome. This complex activates caspase-9, which then activates effector caspases (caspase-3 and caspase-7), leading to the controlled degradation of the cell [94].

It has not yet been definitively clarified whether the control of apoptosis by Bcl-2 family proteins occurs through direct interaction with mitochondria or by influencing apoptosome formation [95]. Some models suggest that Bcl-2 family proteins may inhibit apoptosis not only by preventing the release of cytochrome c from mitochondria but also by interfering with apoptosome formation. This would suggest a role for Bcl-2 proteins in the post-mitochondrial release phase of apoptotic factors [96].

### 4.2. Bcl-2 in Hematological Malignancies

The balance and interaction between anti-apoptotic and pro-apoptotic proteins determine whether a cell survives or dies. This balance is often disrupted in hematological malignancies, with overexpression of anti-apoptotic proteins conferring resistance to apoptosis and allowing uncontrolled proliferation of cancer cells [97].

The overexpression of Bcl-2 is particularly evident in leukemias, lymphomas, especially follicular lymphoma (FL) and chronic lymphocytic leukemia CLL), as well as in some solid tumors, such as breast cancer, lung cancer, and melanoma [98,99]. Along with Bcl-2, other anti-apoptotic proteins, such as Mcl1 and Bcl-xL, play a critical role in the survival of cancer cells in other hematological malignancies, such as acute myeloid leukemia (AML) and multiple myeloma (MM) [100]. Conversely, pro-apoptotic proteins (such as BAX and BAK) may be mutated or dysfunctional in tumors, further increasing the cancer cells’ ability to resist programmed cell death [101].

The relationship between Bcl-2 and Notch proteins plays a significant role in cellular survival mechanisms and therapeutic resistance in various cancer types. In MM, Notch signaling supports tumor cell survival by interacting with Bcl-2 to inhibit apoptosis. Recent studies have shown a notable reduction in myeloma cell proliferation, highlighting a synergistic antitumor effect that could offer a promising therapeutic option for this malignancy [102].

In the context of T-ALL, the block in the maturation pathway of thymocytes at an immature CD4^−^/CD8^−^ double-negative (DN) stage is often caused by mutations leading to hyperactivation of the Notch signaling pathway. This results in the nuclear translocation of the intracellular domain (N-ICD), which acts as a transcription factor and activates numerous pro-oncogenic genes [4]. Concurrently, the expression of apoptosis-regulating proteins dynamically changes during the various phases of thymocyte development to maintain a balance between cell survival and the elimination of inadequately selected cells. Notably, Bcl-2 is more highly expressed in early T-cell progenitors (ETP), and its expression decreases as T cells progress in maturation, reaching the lowest level in DN3-DN4 subsets. This reduction likely facilitates the elimination of thymocytes that fail to successfully rearrange their TCR (T Cell Receptor) genes or meet developmental checkpoints [103]. In contrast, the expression level of Bcl-xL opposes that of Bcl-2, increasing from the ETP to DN3 stage. This upregulation may help thymocytes to survive through the rigorous β-selection process, which ensures only cells with functional pre-TCR complexes continue development. It then gradually decreases after the DN3 stage, reaching lower levels in CD4^+^ or CD8^+^ T cells [103].

At the end of maturation, Bcl-2 levels remain elevated to allow migration to peripheral tissues; this also maintains T cells in a quiescent state, ready to act if appropriately signaled [103].

In T-ALL, the Notch signaling pathway influences Bcl-2 regulation; specifically, Notch hyperactivation leads to the transcriptional upregulation of several anti-apoptotic proteins, including Bcl-2, Bcl-xL, and Mcl1, while down regulating effector proteins such as BAX and BAK. This can also occur through downstream intermediates of the Notch signaling pathway, such as c-MYC [104]. This allows leukemic cells to evade apoptotic signals and to proliferate. The sustained high expression of Bcl-2 confers resistance to antitumor treatments, as these primarily act by inducing apoptosis as a mechanism to eliminate cancer cells [105]. The expression of genes of the anti-apoptotic Bcl-2 family is particularly varied in T-ALL patients; therefore, inhibitors targeting proteins encoded by these genes have variable effects on the various T-ALL cell lines [106]. In this regard, the novel Bcl-2/Bcl-xL dual inhibitor (NWP-0476) was effective against both subtypes of T-ALL (ETP-ALL and typical T-ALL). The authors suggest that their combined action with tyrosine kinase inhibitors (dasatinib and ponatinib) might be effective against relapsed T-ALL [107].

## 5. BH3-Mimetics in T-ALL

As the overexpression and the role of Bcl-2 family proteins contributes to regulating proliferation and cell survival, BH3 mimetics inhibitors are emerging as promising agents against hematological malignancy [108].

BH3 mimetics are engineered peptide inhibitors derived from the BH3 domain of pro-apoptotic proteins, whose function is based on the interaction with the hydrophobic pocket of the anti-apoptotic Bcl-2 proteins, thus allowing BAX/BAK to form the mitochondrial pore complex (MPC) and activate apoptosis [109]. BH3-mimetic drugs can be divided into two categories. Pan-inhibitors have been generated to preferentially bind more than one pro-survival protein, but they may show non-specific off-target effects ([110] and references therein). For this reason, a second category of specific inhibitors has been developed [110], having the advantage of more targeted action; despite this, inhibitors in this category may be less effective if the expression of other Bcl-2 family proteins can compensate for their function. Some BH3 mimetics have been validated and entered clinical trials, but some others have been discontinued due to severe cytotoxic effects, incorrect non-specific protein interactions, or low bioavailability.

### 5.1. ABT-737

ABT-737 was the first BH3-mimetic drug designed by targeting the binding hydrophobic pocket of Bcl-2, Bcl-xL and the Bcl-W BH3 domain, but has low binding efficiency to Mcl1 [108]. In ALL human cell lines and pediatric xenografts, ABT-737 treatment alone induced cell death via mitochondrial apoptosis. This was marked by an increase in hypophosphorylated BID, which binds to and inhibits the anti-apoptotic proteins Bcl-2 and Bcl-xL. BID was further cleaved into its active truncated form, tBID, and triggered by enhanced activation and cleavage of caspases 8, 3, and 9 [111]. However, resistance against ABT-737 has been reported in several preclinical models of different hematological diseases, mainly due to the expression of Mcl1 [108,109,112]. Furthermore, the poor bioavailability and low solubility of ABT-737 seems to be a major obstacle to its further use in clinics [109].

### 5.2. ABT-263 (Navitoclax)

Navitoclax, or ABT-263, is a developed version of ABT-737, with improved oral bioavailability. It is also a pan-inhibitor that targets Bcl-2, Bcl-xL, and Mcl1, with a higher affinity for Bcl-2 (Ki < 0.010 nM) [108]. An in vivo study demonstrated that ABT-263 has excellent therapeutic activity, inducing tumor regressions in 19 out of 31 ALL xenografts. Among these, it has shown a particular efficacy against T-ALL and ETP-ALL xenografts derived from pediatric patients, highlighting the potential of ABT-263 in the treatment of the most aggressive and immature subtype of this disease [113]. Despite promising results in both preclinical and clinical studies, it was associated with dose-limiting thrombocytopenia due to the inhibition of Bcl-xL, which is important for platelet survival ([114]-NCT00481091 [115]).

### 5.3. ABT-199 (Venetoclax)

Venetoclax (ABT-199) was developed as a high-affinity Bcl-2 antagonist (Ki < 0.010 nM) and lower Bcl-xL binding, seeking to overcome the side effects associated with ABT-737 and Navitoclax treatments [109]. In 2016, it was approved by the US Food and Drug Administration (FDA) for first line treatment of R/R CLL, AML, and small lymphocytic lymphoma (SLL) [116]. In vitro studies on a panel of human T-ALL cell lines revealed a modest response to Venetoclax [117]. Resistance in some T-ALL cell lines may be due to high levels of Bcl-xL, as ABT-199 selectively targets Bcl-2 and not Bcl-xL. The Loucy cell line, derived from an immature ETP leukemia, demonstrated high sensitivity to Bcl-2 inhibition, with an IC50 of 13.9 nM [23]. A study investigating the correlation between Venetoclax sensitivity and tumor immunophenotype in two Lck-Lmo2 mouse models identified a 100-fold difference in drug sensitivity between tumors with immature and mature T-cell phenotypes. The immature T-cell tumor, with high Bcl-2 expression, showed significantly greater sensitivity to ABT-199 compared to the mature tumor [23]. In addition, samples of pediatric and adult T-ALL cases, particularly those with an ETP-ALL phenotype, collected from patients enrolled on a Clinical Oncology Group (COG) clinical trial, displayed Bcl-2 dependence [118]. Preclinical trials using xenograft models derived from ETP-ALL patients showed that ABT-199 treatment markedly reduced tumor burden in the bone marrow, spleen, and peripheral blood [118].

One of the most significant side effects associated with ABT-199 is tumor lysis syndrome (TLS), which can occur following the massive activation of apoptosis in tumor cells [108]. Furthermore, mature forms of T-ALL may be less sensitive to Bcl-2 inhibition, limiting the applicability of ABT-199 to specific subgroups of the disease [23].

### 5.4. IS21

Despite progress in treatments, ongoing research continues to explore the development of new pan-inhibitors to enhance the effectiveness of therapies. IS20 and IS21 are two pan-inhibitors that have shown pro-apoptotic effects both in vitro and in vivo, with increased selectivity for tumor cells [99]. Molecular docking analyses have shown that IS21 binds to Bcl-2 in a mode comparable to ABT-199 yet remains unaffected by mutations that confer resistance to ABT-199. Furthermore, IS21 has more favorable ADMET (Adsorption, Distribution, Metabolism, Excretion) properties than ABT-199, making it a promising candidate for drug development [99]. IS21 has been shown to reduce viability in four human T-ALL cell lines with a different Notch genetic background, TALL1, Loucy, and Molt3, which are characterized by high levels of the Bcl-2 protein, while being less effective on the GSI-resistant Jurkat cell line, with low Bcl-2 levels [119].

### 5.5. BH3-Mimetics in Combination Therapy

Despite the promising efficacy of BH3-mimetics as single agents in T-ALL, the combined use of BH3 mimetics with other therapies, such as chemotherapy, may offer a synergistic approach that could enhance antitumor activity, especially in R/R T-ALL, for which very few drugs are available.

Treatment of T-ALL cell lines with Idasanutlin, an MDM2 inhibitor, showed a moderate induction of apoptosis, mainly through the upregulation of BAX, a pro-apoptotic protein [120]. However, when combined with ABT-263, a strong p53-dependent synergistic activity was observed in PDX T-ALL lines with NOTCH1 and/or FBXW7 mutations [120].

In another study, the combined treatment of Venetoclax with BET bromodomain inhibitors (JQ1) showed a more powerful synergistic effect in inducing apoptosis in human T-ALL cell lines, Loucy and ALL-SIL. Treatment with JQ1 upregulates the expression of the Bcl-2L11 gene, which encodes the pro-apoptotic protein BIM. Increased BIM expression was needed to interact and bind Bcl-2 and make cells more sensitive to the effect of Venetoclax [121].

When combined with chemotherapeutics such as doxorubicin, asparaginase, and dexamethasone, Venetoclax can amplify the cytotoxic effect on T-ALL tumors, especially in those subtypes that show low IC50 values, suggesting a high sensitivity to the drug [23,119]. This combined approach not only increases the efficacy of the treatment but could also reduce the side effects of chemotherapy. Furthermore, the combination of ABT-199 with these therapeutic agents could prevent or delay the onset of resistance to treatment, a common problem in anticancer therapies [116].

Furthermore, IS21 treatment with doxorubicin or vincristine treatment increased apoptosis in T-ALL human cell lines, especially in Jurkat cells, which, as mentioned above, have been shown to be the most resistant to single treatments with the inhibitor due to low levels of Bcl-2 [119].

Several clinical trials are ongoing evaluating the effectiveness of Bcl-2 inhibition in combination with conventional chemotherapy agents in T-ALL (NCT00501826 [119]; NCT03808610, NCT03504644 [122]). In a small cohort of patients with R/R T-ALL, 60% achieved a bone marrow response to combination treatment with the addition of Venetoclax compared to single treatments with nelarabine (31–36%) and intensive chemotherapy (20–40%). Notably, two patients with ETP-ALL were in remission at the last follow-up examination [122].

A retrospective multicenter analysis recently reported on R/R adult ALL patients, including eight ETP-ALL and twenty-two T-ALL cases, treated with venetoclax alone or in combination with low doses of navitoclax [123]. The authors describe an overall response rate higher in the venetoclax-navitoclax cohort than in the venetoclax single-agent cohort, supporting the synergistic effect of the dual inhibition of Bcl-2 and Bcl-xL. Patients who received associated chemotherapy did not show superior response rates [123]. Additionally, no early toxic fatal events or unexpected and dose-limiting toxicities were observed. Ongoing clinical trials with venetoclax and navitoclax are under investigation with either chemotherapy NCT05268003, NCT03319901) or in frontline settings (NCT05386576, NCT03319901) [123].

## 6. Notch Inhibitors and BH3 Mimetics in T-ALL

Looking to the future and innovative therapeutic strategies, we report preclinical data combining the BH3-mimetics with γ-secretase inhibitors (GSI) or calcium pump (SERCA) inhibitors that could potentially offer new approaches for treating T-ALL.

### 6.1. Combining BH3 Mimetics with γ-Secretase Inhibitors (GSIs)

Only three reports [102,124,125] explored the combined effect of GSI and ABT-737 in solid and hematological malignancies, with only one study examining ABT-199 with GSI specifically in breast cancer [126]. In multiple myeloma (MM), the combination of ABT-737 and GSIs leads to BAK and BAX activation and consequently to cytochrome c release, enhancing the synergistic effect of the drugs and suggesting this combination as a potential therapeutic strategy for MM patients.

As far as we know, the only case reported in the literature shows that GSI (CompE) treatment made previously GSI-resistant T-ALL cell lines 10- to 100-fold more resistant to ABT-263. Even in GSI-sensitive cell lines, GSI treatment reduced sensitivity to this BH3-mimetic. This effect was further validated with two other γ-secretase inhibitors, MRK-003 and RO-4929097. The increased resistance in GSI-treated cell lines was linked to elevated levels of Mcl1. Since ABT-263 has a low binding affinity for Mcl1, this was significant. Further investigation revealed that GSI treatment activated mTORC1, which in turn increased Mcl1 levels, thus requiring the use of an mTORC1-inhibitor to enhance ABT263 sensitivity [117].

Additionally, the recently developed Mcl1 specific inhibitor, S63845, has shown promising results by actively killing T-ALL cell lines as a single agent. The synergistic inhibition of Mcl1 and Bcl-2, in a combination treatment of the same cell lines with S63845 and Venetoclax, resulted in potentiated apoptotic cell death [127]. In silico studies indicate that simultaneously inhibiting these pathways can provide an additive benefit. This highlights the potential of using Venetoclax and S63845 together and suggests that their combination with GSIs may further improve therapeutic outcomes by effectively targeting Notch signaling in breast cancer stem cells [126].

### 6.2. Combining BH3 Mimetics with SERCA Inhibitors

Dual targeting on the apoptotic resistance machinery of tumor cells with anti-apoptotic Bcl-2 family proteins and SERCA proteins has been suggested in the past by experiments performed in leukemic cell lines, including Jurkat cells [128]. Recently, Pagliaro L. et al. investigated a new treatment strategy that combines Notch inhibition using the SERCA inhibitor CAD204520 together with the first-line treatment Venetoclax, and ibrutinib (EMA and FDA-approved), mainly in Notch1-mutated CLL patients [129]. This would suggest the potential to exploit this approach in T-ALL or in ETP-TALL patients due to their high levels of Bcl-2 and the aggressive nature of the disease.

These early data suggest the need for further in vitro/in vivo studies to unveil the underlying molecular mechanisms, and that Bcl-2 expression and BH3-mimetics response depend on T-ALL genetics and immunophenotype, meaning that they could be exploited as biomarkers for therapy.

## 7. Future Perspectives

Notch and Bcl-2 are critical factors in modulating early steps in T-cell development from ETP to pro-T and then to Pre-T cells [25]. Conversely, based on the increased expression of Bcl-xL in more mature T-cells from Pre-T to DP cells and the dependency of typical T-ALL on this Bcl-2 family member, BH3 mimetics profiling [123] and the incorporation of cytogenetic analysis focused on Notch [130] in primary samples could serve as laboratory predictive tools to design the combined therapy with Notch inhibitors considering the maturation stage arrest of the T-ALL.

Although BH3 inhibitors have demonstrated potential in T-ALL therapy, particularly when used in combination treatments, further research is necessary to optimize their effectiveness. Targeting the Notch signaling pathway together with anti-apoptotic proteins of the Bcl-2 family, both intimately associated with drug resistance, may be one of the strategies to improve the treatment of T-ALL (Figure 2) since multiple pathways contribute to it. In this regard, the recent comprehensive genomic characterization of T-ALL will provide valuable insights. Although promising, limitations to this study are posed by questions such as clinical validation, complexity of therapy resistant mechanisms, and the need for longitudinal studies to test the long-term efficacy of Notch inhibitors and BH3 mimetics to prevent and more to achieve long-lasting control over T-ALL. Based on the reports discussed so far, the dual targeting of Bcl-2 family proteins and Notch could empower the sensitivity and the outcome when combined with standard protocols. How such a combination will be tolerated in terms of toxicity, side-effects, and the feasibility of long-term treatment will need to be carefully assessed.

In the future, developing new and well-tolerated drugs against Notch, and more specifically for Bcl-2 family proteins, could improve targeted therapy in T-ALL.

## Figures and Tables

**Table 1:** Summary of *γ*-secretase inhibitors (GSI) used in preclinical and clinical T-ALL studies. * Clinical trial identifier code (clinicaltrials.gov).

Drugs Name	Targeted Pathways	Clinical Trials			Effects	References
		Phase	Year	Status		
PF-03084014	NF-kB phosphorylation, Caspase-3 cleavage and PARP resulting in decreased Notch2 activity compared to other Notch receptors	Phase I study (NCT00878189) *	2009	Completed	No conclusive antileukemic effect in R/R T-ALL patients, although diarrhea was not a dose-limiting toxicity	[43,44]
RO-4929097	Notch processing, lowering Notch and Hes1 expression. It does not stop cell proliferation or cause apoptosis. Cells appear less transformed, flatter, and grow more slowly	Phase I/II study (NCT01088763) *	2010	Terminated	Efficacy has not been established in T-ALL patients. Frequent toxic reactions include diarrhea, nausea, fatigue, hypophosphatemia, vomiting, rash, and decreased appetite	[45,46]
BMS-906024	Selective on Notch1 cleavage, while sustaining Notch3 cleavage with no signaling shut down. Deeper studies of the mechanism are required	Phase I study (NCT01363817) *	2011	Completed	At low concentrations, it selectively inhibits Notch1 cleavage while supporting Notch3 cleavage, therefore, signaling is not inhibited and there is no benefit	[47]
MRK-560	Selective on PSEN1-containing GS complexes, while leaving PSEN2- containing complexes untargeted. Decreased mutant Notch1 processing and cell cycle arrest. T-ALL primary samples express only PSEN1-containing GS complexes	Preclinical Studies (patient-derived xenografts)	―	―	No gastrointestinal toxicity or T-cell development defects, partially relying on preserved Notch function due to equivalent expression of PSEN1 and 2 in these contexts. PSEN2-knock-out mice highlighted PSEN2’s protective role. A potential therapeutic strategy for safe and effective targeting of T-ALL	[48]
